# Differential Responses of the Antioxidant System of Ametryn and Clomazone Tolerant Bacteria

**DOI:** 10.1371/journal.pone.0112271

**Published:** 2014-11-07

**Authors:** Leila Priscila Peters, Giselle Carvalho, Paula Fabiane Martins, Manuella Nóbrega Dourado, Milca Bartz Vilhena, Marcos Pileggi, Ricardo Antunes Azevedo

**Affiliations:** 1 Departamento de Genética, Escola Superior de Agricultura Luiz de Queiroz, Universidade de São Paulo, Piracicaba, Brazil; 2 Departamento de Biologia Estrutural, Molecular e Genética, Universidade Estadual de Ponta Grossa, Ponta Grossa, Brazil; Universidad Pablo de Olavide, Centro Andaluz de Biología del Desarrollo-CSIC, Spain

## Abstract

The herbicides ametryn and clomazone are widely used in sugarcane cultivation, and following microbial degradation are considered as soil and water contaminants. The exposure of microorganisms to pesticides can result in oxidative damage due to an increase in the production of reactive oxygen species (ROS). This study investigated the response of the antioxidant systems of two bacterial strains tolerant to the herbicides ametryn and clomazone. Bacteria were isolated from soil with a long history of ametryn and clomazone application. Comparative analyses based on 16S rRNA gene sequences revealed that strain CC07 is phylogenetically related to *Pseudomonas aeruginosa* and strain 4C07 to *P. fulva*. The two bacterial strains were grown for 14 h in the presence of separate and combined herbicides. Lipid peroxidation, reduced glutathione content (GSH) and antioxidant enzymes activities were evaluated. The overall results indicated that strain 4C07 formed an efficient mechanism to maintain the cellular redox balance by producing reactive oxygen species (ROS) and subsequently scavenging ROS in the presence of the herbicides. The growth of bacterium strain 4C07 was inhibited in the presence of clomazone alone, or in combination with ametryn, but increased glutathione reductase (GR) and glutathione S-transferase (GST) activities, and a higher GSH concentration were detected. Meanwhile, reduced superoxide dismutase (SOD), catalase (CAT) and GST activities and a lower concentration of GSH were detected in the bacterium strain CC07, which was able to achieve better growth in the presence of the herbicides. The results suggest that the two bacterial strains tolerate the ametryn and clomazone herbicides with distinctly different responses of the antioxidant systems.

## Introduction

Pesticides are powerful tools in modern agriculture to minimize economic losses caused by weeds, insects and diseases, and to ensure adequate food production [1], [2]. It is estimated that nearly 3 billion tons of pesticides are released into the environment each year [3], more than 35% of which are herbicides [4]. The intensive use of these xenobiotics in agroecosystems can result in the contamination of water and soils [5]–[7].

The herbicides ametryn and clomazone are widely employed in crops such as sugarcane, soybeans, corn, cotton, and are often detected in the environment [8]–[11]. In plants, ametryn toxicity is related to the blockage of the electron transport chain binding specifically to D1 proteins of photosystem II, thereby preventing photosynthesis [12]. In contrast, the mode of action of clomazone consists of inducing lipid peroxidation in cells by blocking carotenoid synthesis [13], [14]. These two herbicides in the soil can affect microbial activity and induce a selection pressure, which in turn allows the identification of tolerant microorganisms [15],[16].

Previous studies have shown that many herbicides are redox-cycling agents able to alter the aerobic metabolism of microorganisms culminating in an oxidative stress condition [17]–[19]. This process induced by herbicides in bacteria results in the increased production and subsequent accumulation of reactive oxygen species (ROS), such as the superoxide radical (O_2_^•−^), hydrogen peroxide (H_2_O_2_) and hydroxyl radical (OH^•^) [17]. These products of aerobic cell metabolism are toxic and may lead to enzyme inactivation, protein denaturation, lipid peroxidation and DNA mutation [20], [21]. Therefore, any excess ROS that is produced has to be eliminated if a microbe is to survive [22].

Many bacteria can increase the rate of synthesis and accumulate non-enzymatic antioxidant compounds in response to excessive production of ROS (e.g. reduced glutathione (GSH) and ascorbic acid), as well as increase the activity of antioxidant enzymes [18], [23]. The enzymes superoxide dismutase (SOD, EC 1.15.1.1) and catalase (CAT, 1.11.1.6) play crucial roles in the detoxification process of O_2_^•−^ and H_2_O_2_, respectively [24]–[26]. Furthermore, the enzyme glutathione reductase (GR, EC 1.6.4.2) carries out the reduction of oxidized glutathione (GSSG), which is a fundamental reaction for maintaining the homeostasis between GSH/GSSG levels [27]. GSH is an antioxidant capable of directly neutralizing OH^•^ [28] and thus, is considered a key compound, in the stress tolerance process [29]. Glutathione S-transferase (GST, EC 2.5.1.18) is another important enzyme that is required for the degradation of pollutants, since it is primarily involved in cellular detoxification and redox biochemical mechanisms [30], [31].

In this study we have examined the effects of the herbicides ametryn and clomazone on the antioxidant stress responses of two bacteria isolated from agricultural soils, previously treated with herbicides.

## Materials and Methods

### Ethics statement

The bacteria used in this work were isolated from soil samples collected in Fazenda Areão, Escola Superior de Agricultura Luiz de Queiroz (47°38′00″W; 22°42′30″S), Piracicaba, São Paulo State, Brazil. The location is an experimental area of the University and no specific permissions were required for sampling soils in this location. This field sampling did not involve or cause any harm to endangered or protected species.

### Herbicides

Relevant characteristics of the two herbicides, ametryn (2-ethylamino-4-isopropylamino-6-methyl-thio-s-triazine) and clomazone (2-(2-chlorophenyl) methyl-4,4-dimethyl-3-isoxazolidinone), are listed in Table 1. Ametryn, which is a selective herbicide (Gesapax 500, Ciba Geigy) applied at 6 L ha^−1^ (3 kg ha^−1^) to control narrow leaved weeds and broad leaved weeds, was used at 500 g L^−1^ (active ingredient-ai). Clomazone, which is a selective herbicide (Gamit 360 CS) that can be applied at a recommended dose of 1.8 L ha^−1^ (650 g ai ha^−1^) in sugarcane and maize, was used at 360 g ai L^−1^. However, ametryn and clomazone can be applied in combination in sugarcane at a recommended dose of 1 L ha^−1^ (300 g ai ha^−1^), was used at 300 g ai L^−1^ and 1 L ha^−1^ (200 g ai ha^−1^), was used at 200 g ai L^−1^, respectively.

**Table 1:** Characteristics of the herbicides ametryn and clomazone.

	Ametryn	Clomazone
Manufacture	Syngenta, BASF, Bayer, Servatis, Sipcam Isagro Brasil	FMC Corporation
Agrochemical formulation	500 g ai L^−1^, EC	360 g ai L^−1^, EC
Molecular formula	C_9_H_17_N_5_S	C_12_H_14_ClNO_2_
Molecular weight	227.3	239.7
Vapor pressure	2.74×10^−6 ^mm Hg at 25°C	1.4×10^−4^ mm Hg at 25°C
Water solubility	185 mg L^−1^ at 25°C	1,100 mg L^−1^ at 25°C

### Bacterial strains isolation and growth conditions

The bacteria used in this work were isolated from soil samples collected in Fazenda Areão, Escola Superior de Agricultura Luiz de Queiroz (47°38′00″W; 22°42′30″S), Piracicaba, São Paulo State, Brazil. The soils were classified as Oxisol [32] of medium texture and had a history of ametryn and clomazone applications for five consecutive years.

The initial bacterial isolation was carried out using a plating technique with a serial dilution in 0.85% NaCl at concentrations of 10^−3^ and 10^−5^ inoculated in Minimal Salts medium containing 1.0 g (NH_4_)_2_SO_4_, 1.0 g NaCl, 1.5 g K_2_HPO_4_, 0.5 g KH_2_PO_4_, and 0.2 g MgSO_4_.7H_2_O, per L of distilled water, at 30°C (pH 7.0) [33] in the absence and presence of the two herbicides. The concentrations of 25 mM ametryn, 9 mM clomazone and 20 mM of each herbicide were used based on the recommendations on the spray tank solution for each herbicide (5 g L^−1^ for ametryn, 1.8 g L^−1^ for clomazone and 5 g L^−1^ each, in combination).

The tolerant bacterial strains, CC07 and 4C07, were selected based on faster growth rates (compared to other bacteria isolates) and halo formation observed around the bacterial colony, indicating possible herbicide degradation, as observed by Nie et al. [33] and Martins et al. [34].

The bacterial strains were grown aerobically in nutrient Agar (Biobrás - Brazil) containing 5 g peptone, 3 g yeast extract and 15 g agar per L of distilled water, at 30°C (pH 7.0) both in the absence and presence of the herbicides. The herbicides concentrations were added as described above.

### Bacterial identification

Bacterial DNA was extracted as previously described by Araújo et al. [35] and a partial sequence of the 16S rRNA gene was amplified with primers R1387 [36] and P027F [37]. PCR products were purified and sequenced with primers R1387, 519R and P027F for 16S rRNA (MegaBACE 1000). The sequences of the bacterial strains CC07 and 4C07 were retrieved from databases and used for alignment and phylogeny analyses [38], [39] with MEGA 4.0 software package [40] based on the maximum parsimony (MP). The sequences obtained were deposited in GenBank^T^ under the accession numbers JX109938 and JX109935 for strains CC07 and 4C07, respectively.

### Growth determination

Bacterial growth was monitored by measuring the number of colony-forming units (CFUs) mL^−1^ as described by Sangali and Brandelli [41]. Cultures inoculated with 0.1% of the original (Absorbance  = 1.0 at 600 nm) were grown in 250 mL Erlenmeyer flasks containing 50 mL of nutrient medium and incubated in the dark on a rotary shaker (140 rpm) at 30°C for 14 h. The bacterial suspension was diluted to 10^−6^ in a saline solution containing 0.85% NaCl and then homogenized. At 2 h intervals the samples (20 µL) were loaded in triplicate for each treatment onto nutrient agar plates, which were further incubated at 30°C for 24 h. At the end of this period the CFUs were determined.

### Physiological and biochemical measurements

#### Lipid peroxidation

Lipid peroxidation was determined by estimating the content of thiobarbituric acid reactive substance (TBARS) following the method of Heath and Packer [42]. Malondialdehyde (MDA) was monitored by measuring at 535 and 600 nm in a Perkin Elmer Lambda 40 spectrophotometer, and the concentration was calculated using an extinction coefficient of 155 mM^−1^ cm^−1^.

#### Quantification of reduced glutathione (GSH)

Bacterial cells (100 mg) were homogenized in 1.5 mL of 5% sulfosalicylic acid in a mortar and pestle at 4°C. The homogenate was centrifuged at 12,000×*g* for 20 min at 4°C. The content of GSH and GSSG was determined as described by Anderson [43] at 25°C in a mixture consisting of 1.75 mL 100 mM potassium phosphate buffer (pH 7.5) containing 0.5 mM ethylenediaminetetraacetic acid (EDTA) and 100 µL of 3 mM 5,5′-dithiobis(2-nitrobenzoic acid) (DTNB). The reaction was started by the addition of 250 µL of the bacterial cell homogenate. After 5 min, the absorbance for the determination of GSH was read at 412 nm using a spectrophotometer. A standard curve was prepared with known concentrations of GSH and the results were expressed in nmol g^−1^ FW.

#### Enzyme extraction and protein determination

Cultures were centrifuged at 12,000×*g* for 20 min at 4°C and the pellets macerated with liquid nitrogen in a mortar with a pestle. The extracts were homogenized (5∶1, buffer volume: fresh weight) in 100 mM potassium phosphate buffer (pH 7.5) containing 1 mM EDTA, 3 mM DL-dithiothreitol (DTT) and 5% (w/w) polyvinylpolypyrrolidone [44]. The homogenates were centrifuged at 12,000×*g* for 30 min at 4°C and the supernatants were stored in separate aliquots at −80°C prior to enzymatic analysis. The concentration of protein was determined by the method of Bradford [45] using bovine serum albumin as standard.

#### Polyacrylamide gel electrophoresis (PAGE)

Electrofphoretic analyses of antioxidant enzymes were carried out under non-denaturing condition in 12% polyacrylamide gels as described by Gratão et al. [44]. For denaturing SDS-PAGE, the gels were rinsed in distilled deionized water and incubated overnight in 0.05% Coomassie blue R-250 in a water/methanol/acetic acid 45∶45∶10 (v/v/v) solution and destained by successive washings in the same water/methanol/acetic acid 45∶45∶10 (v/v/v) solution.

#### SOD activity staining

SOD activity staining was carried out as described by Garcia et al. [46]. After non-denaturing PAGE separation, the gel was rinsed in distilled deionized water and incubated in the dark in 50 mM potassium phosphate buffer (pH 7.8) containing 1 mM EDTA, 0.05 mM riboflavin, 0.1 mM nitroblue tetrazolium, and 0.3% N,N,N′,N′-tetramethylethylenediamine. One unit of bovine liver SOD (Sigma, St. Louis, USA) was used as a positive control of activity. After 30 min, the gels were rinsed with distilled deionized water and then illuminated in water until the development of achromatic bands of SOD activity on a purple-stained gel. SOD isoenzyme characterization was performed as described by Azevedo et al. [47]. Briefly, SOD isoenzymes were distinguished by their sensitivity to inhibition by 2 mM potassium cyanide and 5 mM hydrogen peroxide. The relative intensities of the stained bands were determined by an ImageScanner III (GE Healthcare, Little Chalfont, UK) and the ImageQuant™ TL software (GE Healthcare, Uppsala, Sweden).

#### CAT activity staining

CAT activity following non-denaturing PAGE was determined as described by Boaretto et al. [48]. Gels were incubated in 0.003% hydrogen peroxide (H_2_O_2_) for 10 min and subsequently in a 1% (w/v) ferric chloride (FeCl_3_) and 1% (w/v) potassium hexacyanoferrate III (K_3_Fe(CN_6_) solution for additional 10 min. One unit of bovine liver CAT (Sigma, St. Louis, USA) was used as a positive control.

#### GR activity staining

GR activity following non-denaturing PAGE was determined as described by Gomes-Junior et al. [49]. The gels were rinsed in distilled deionized water and incubated in the dark for 30 min at room temperature in the reaction solution contained 250 mM Tris (pH 7.5), 0.5 mM 3-(4,5-dimethyl-2-thiazolyl)-2,5-diphenyl-2H-tetrazolium bromide (MTT), 0.7 mM 2,6-dichloro-*N*-(4-hydroxylphenyl)-1,4-benzoquinoneimine sodium salt (DPIP), 3.4 mM GSSG (oxidized glutathione) and 0.5 mM NADPH. One unit of bovine liver GR (Sigma, USA) was used as a positive control of activity.

#### GR total activity determination

Total GR activity was assayed as described by Gratão et al. [44] at 30°C in a mixture consisting of 1.7 mL 100 mM potassium phosphate buffer (pH 7.5) containing 1 mM 5,5′-dithiobis(2-nitrobenzoic acid) (DTNB), 1 mM GSSG and 0.1 mM NADPH. The reaction was started by the addition of 50 µL of protein extract. The rate of reduction of oxidized glutathione was followed in a spectrophotometer by monitoring the change in absorbance at 412 nm for 1 min. GR activity was expressed as µmol min^−1^ mg^−1^ protein.

#### GST total activity determination

GST activity was assayed spectrophotometrically at 30°C in a mixture containing 900 µL 100 mM potassium phosphate buffer (pH 6.5), 25 µL 40 mM 1-chloro-2,4-dinitrobenzene (CDNB), 50 µL 1 mM GSH and 25 µL enzyme extract. The reaction mixture was followed by monitoring the increase absorbance at 340 nm over 5 min [50]. GST activity was expressed as µmol min^−1^ mg^−1^ protein.

#### Experimental design and statistical analysis

Total protein content and enzyme activity determinations were conducted on three replicates of each treatment, which were performed in a completely randomized design. The significance of the observed differences was verified by using a one-way analysis of variance (ANOVA) followed by the Tukey's test (p<0.05). All statistical analyses were carried out by using R software (URL http://www.r-project.org).

## Results

### Phylogenetic identity and bacterial growth

**Figure 1:**
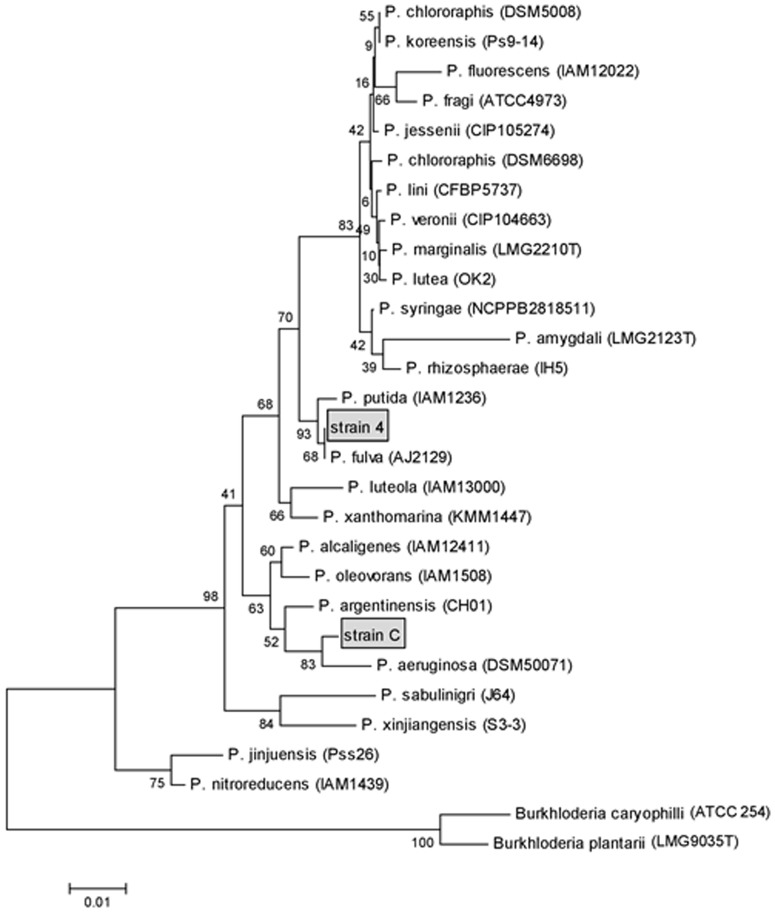
Maximum-parsimony phylogenetic tree constructed from the 16S rRNA gene. A total of 600 bp nucleotide of *Pseudomonas* spp. from RDP database were used. *Burkhloderia cariophilli* and *B. plantarii* served as outgroup. Bootstrap values were 1000 repetitions. Bars indicate the number of evolutionary steps with diverging sequences. Strains CC07 and 4C07 are shown inside the boxes.

An almost-complete 16S rRNA gene sequence (1312 nts) was determined for both the CC07 and 4C07 strains and a phylogenetic tree was built up (Fig. 1). *Burkhloderia cariophilli* and *B. plantarii* were used as outgroups. Comparative analyses based on 16S rRNA gene sequences revealed that the CC07 strain is phylogenetically related to *Pseudomonas aeruginosa*, whereas the 4C07 strain exhibited homology with *P. fulva* (Fig. 1).

**Figure 2:**
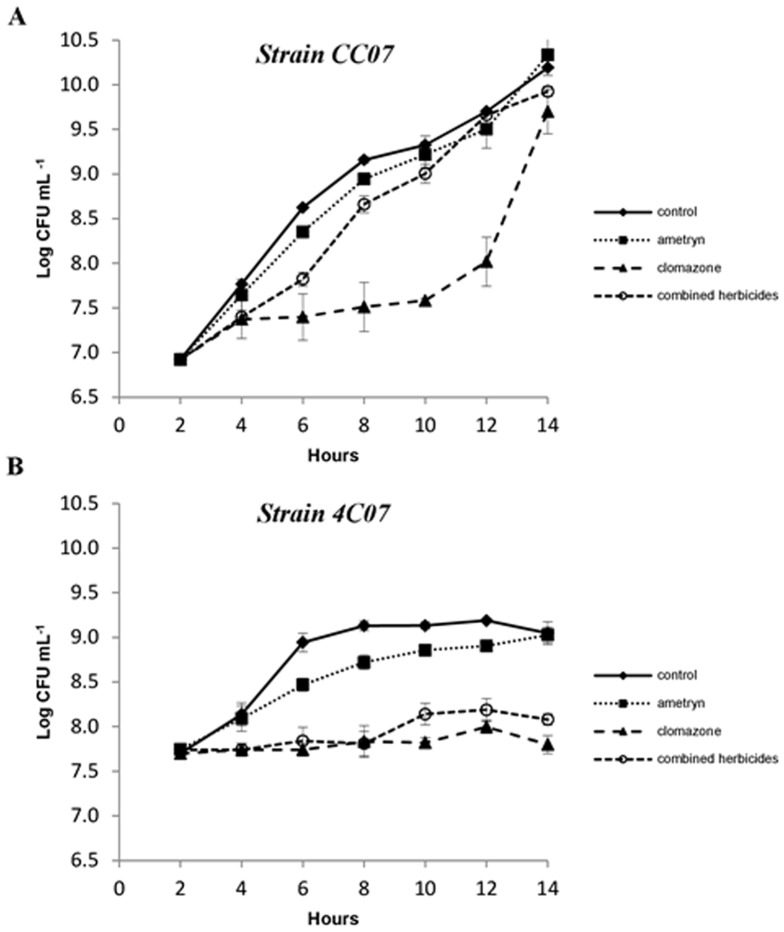
Growth curve of bacterial strains in the presence of 0 mM (control), 25 mM ametryn, 9 mM clomazone and 20 mM of each the herbicide. (A) Strain CC07 and (B) Strain 4C07. Values represent the means from three replicates ±SEM.

The growth of the strains in the presence of the herbicides is shown in Fig. 2. The two strains exhibited very distinct growth curves. Growth of both strains was not greatly affected in the presence of the herbicide ametryn, whilst clomazone strongly inhibited the growth of both strains. Strain CC07 exhibited a long (10 h) lag phase as an adaptation period before the exponential growth (12 h) (Fig. 2A), while strain 4C07 grew only for the first six hours. When the herbicides were used in combination, growth of the strain CC07 was only slightly inhibited, mainly during the early period, whereas for strain 4C07 there was considerable inhibition of growth, similar to that shown by clomazone alone.

### Lipid peroxidation (MDA)

**Figure 3:**
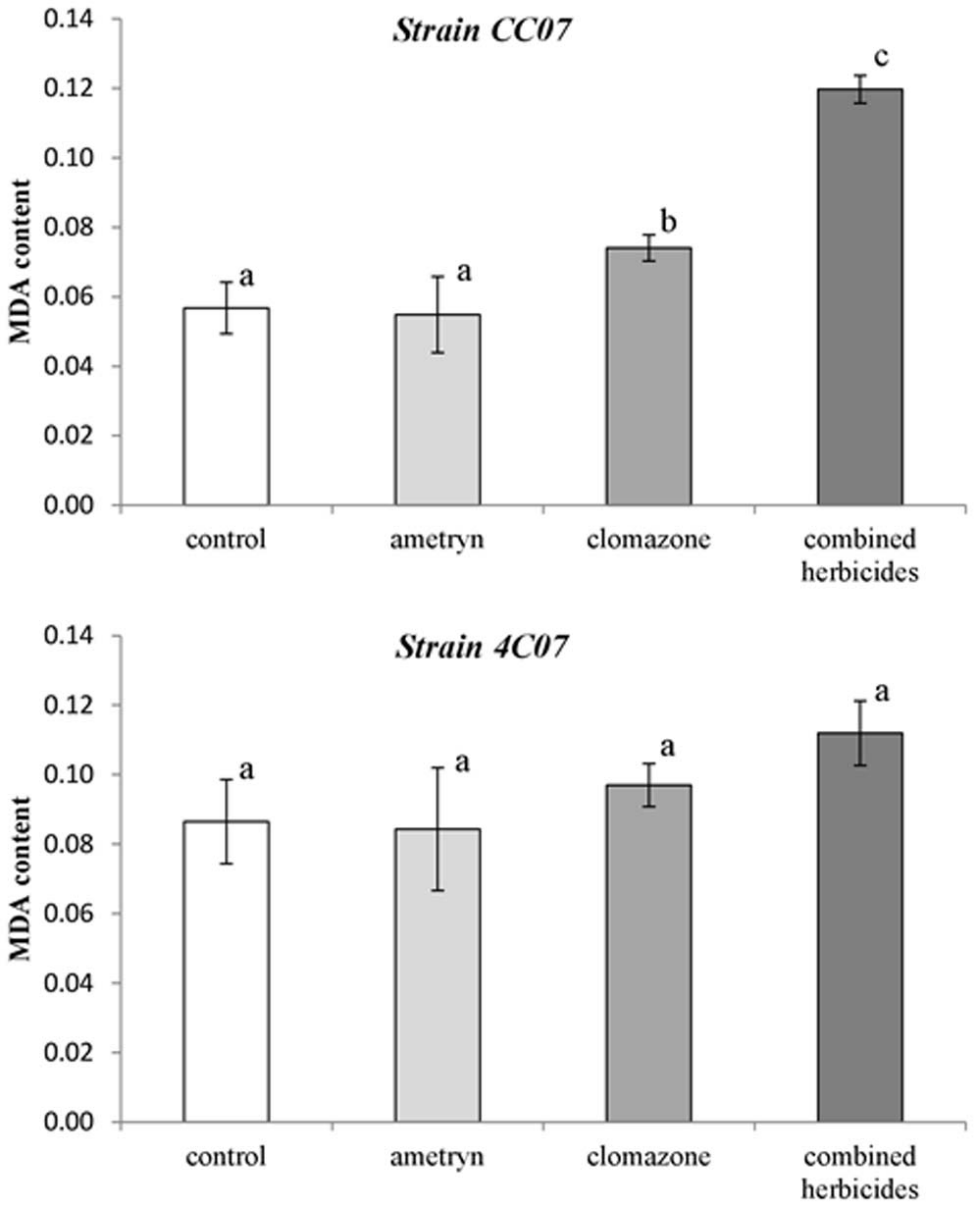
Lipid peroxidation (MDA content) of bacteria exposed to the herbicides. Values of MDA content (nmol g^−1^ fr. wt) represent the means from three replicates ±SEM. Means with different letters are significantly different (*P<*0.05) by one-way analysis of variance (ANOVA) and Tukey's test.

Lipid peroxidation was determined as the MDA content after 14 h of growth. Although similar trends in MDA content were detected in both strains (Fig. 3), statistically there was only a higher MDA content in strain CC07 in the presence of clomazone (30.3%) and when exposed to the combination of the two herbicides (110.7%) (Fig. 3), when compared to growth in the control herbicide-free medium.

### The content of reduced glutathione (GSH)

**Figure 4:**
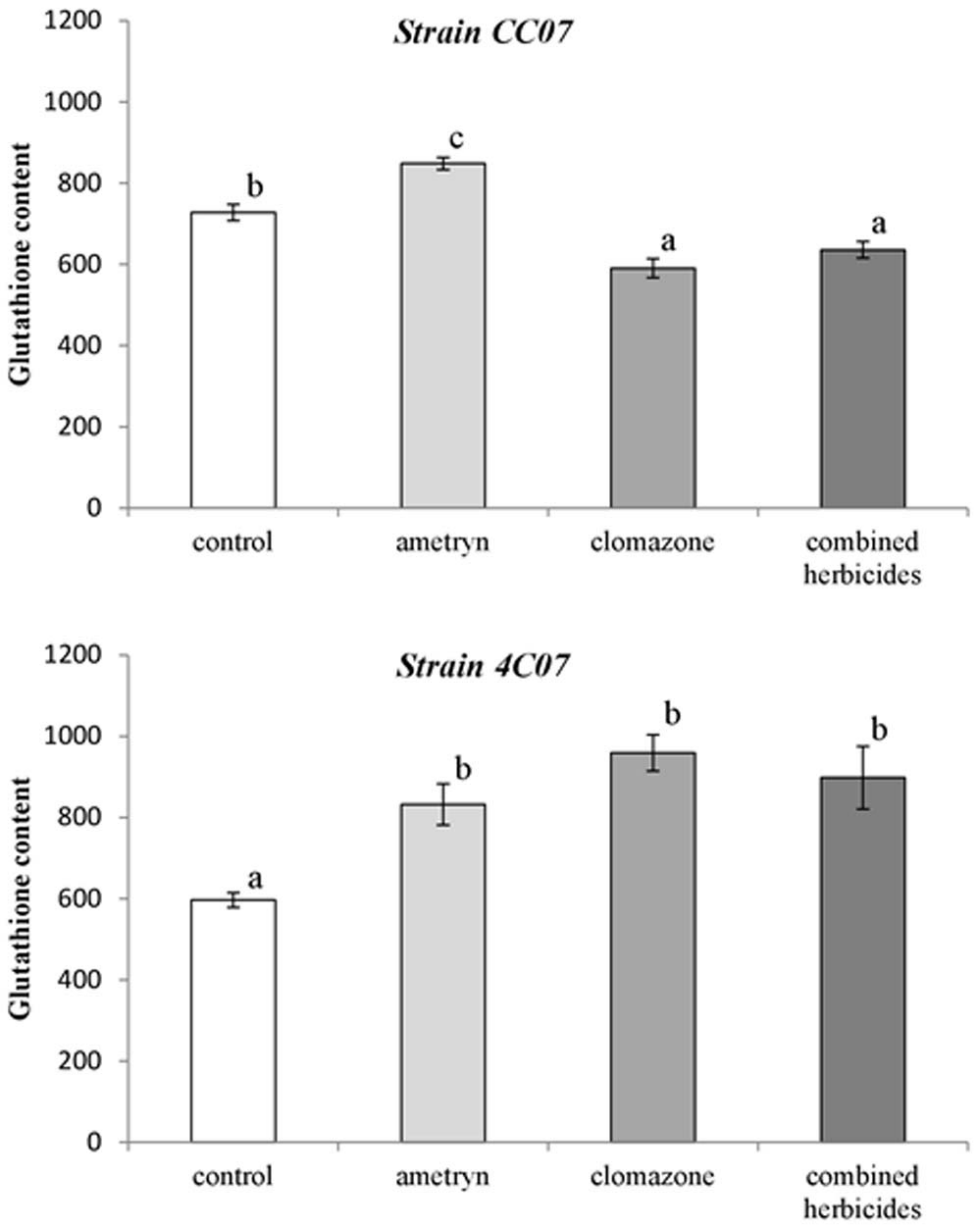
GSH content (nmol g^-1^ fr. wt) of bacteria exposed to the herbicides. Values represent the means from three replicates ±SEM. Means with different letters are significantly different (*P<*0.05) by one-way analysis of variance (ANOVA) and Tukey's test.

Different amounts of GSH were detected in the two bacterial strains, following exposure to the herbicides (Fig. 4). There was a significant increase in the GSH content of CC07 (16.5%) in the presence of ametryn, but GSH was reduced below control levels in the presence of clomazone or the herbicide mixture. Meanwhile in strain 4C07, the GSH content increased, following exposure to all the herbicide treatments (ametryn 39.4%, clomazone 60.7% and the mixture 50.5%), when compared to the untreated control (Fig. 4).

### SDS-PAGE protein profile

**Figure 5:**
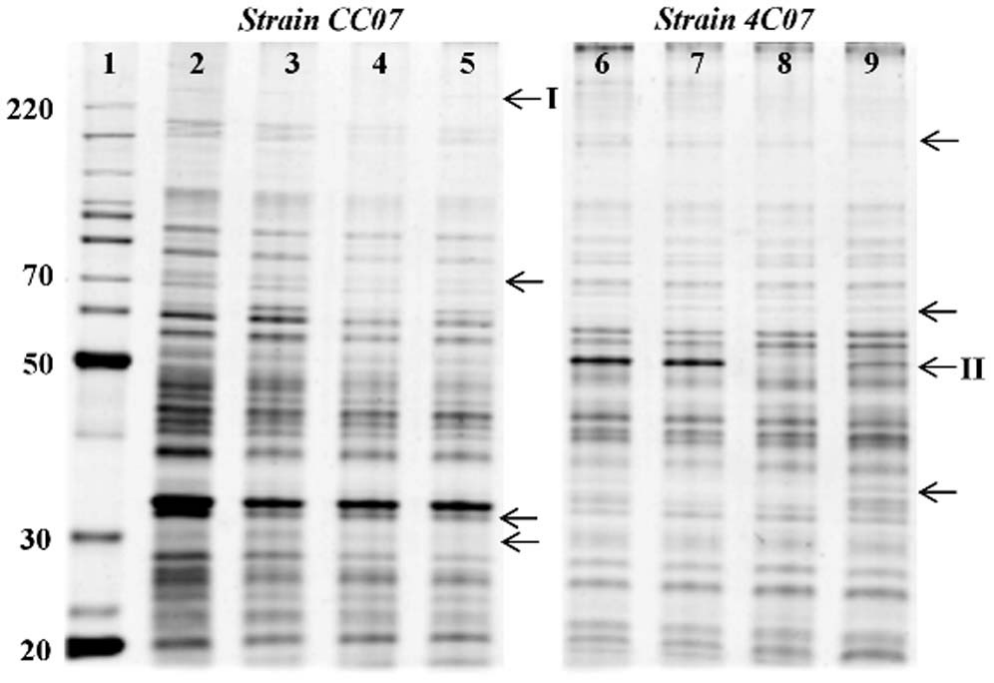
SDS-PAGE protein profiles of bacteria exposed to the herbicides. Lane 1, protein molecular mass markers (220 to 20 kDa). Lanes 2, 3, 4 and 5, strain CC07 grown in the presence of 0 mM (control), 25 mM ametryn, 9 mM clomazone and 20 mM of each the herbicide, respectively. Lanes 6, 7, 8 and 9, strain 4C07 grown in the presence of 0 mM (control), 25 mM ametryn, 9 mM clomazone and 20 mM of each the herbicide, respectively. Arrows indicate selected variations in intensity or appearance/disappearance depending on the treatment tested. I and II indicate protein bands of 225 kDa and 58 KDa that are depleted specifically in the presence of clomazone.

Analysis following SDS-PAGE revealed clearly different protein profiles between the two bacterial strains, both with and without herbicide treatments (Fig. 5). There was a general reduction in intensity of the majority of the protein bands following electrophoresis of extracts of CC07 that had been subjected to herbicide treatment (Fig. 5, lanes 3, 4 and 5). In addition, a number of protein bands varied in intensity or appearance/disappearance, depending on the treatment as indicated by the arrows in Fig. 5. For instance, a 225 kDa protein band (strain CC07) was reduced following treatment with the herbicide clomazone (Fig. 5, lane 4, band I), and a 58 kDa protein band was absent in strain 4C07 exposed to clomazone (Fig. 5, lane 8, band II), and greatly reduced following treatment by both herbicides (Fig.5, lane 9, band II), among other changes (arrows).

### Effects of the herbicides on antioxidant enzymes

**Figure 6:**
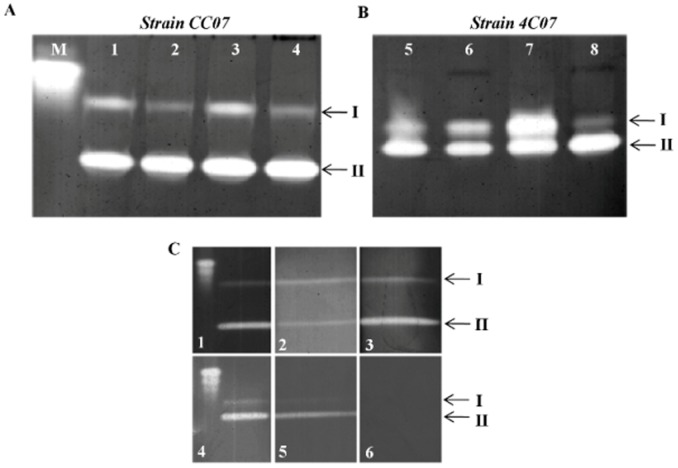
Activity staining for SOD following non-denaturing PAGE of extracts from cultured bacterial cells. (A) First lane is a bovine SOD standard, lanes 1, 2, 3 and 4 are strain CC07 grown in the presence of 0 mM (control), 25 mM ametryn, 9 mM clomazone and 20 mM of each herbicide, respectively. (B) Lanes 5, 6, 7 and 8 are strain 4C07 grown in the presence of 0 mM (control), 25 mM ametryn, 9 mM clomazone and 20 mM of each herbicide, respectively. Arrows indicate sequentially numbered SOD bands (I–II) that are independent of the bacterial strain. (C) Activity staining for SOD of strain CC07 (1, 2 and 3) and strain 4C07 (4, 5 and 6), used for classification of SOD isoenzymes. Lanes 1 and 4, control SOD activity. Lanes 2 and 5, SOD activity with 2 mM potassium cyanide treatment; lanes 3 and 6; 5 mM H_2_O_2_ treatment. Arrows indicate SOD bands that are sequentially numbered (I–II) according to Fig. 6A and B.

There were different responses in the antioxidant enzymes (SOD, CAT, GR and GST) isolated from the two bacterial strains, when treated with the herbicides alone or in combination. SOD activity was determined by activity staining following non-denaturing PAGE (Fig. 6). The analysis revealed that there were two distinct isoenzymes present in each bacterium, but that they had different electrophoretic mobilities (Fig. 6A and B). Both of the SOD isoenzymes isolated from strain CC07 were characterized as Mn/SODs (Fig. 6A, SOD I and II, lanes 1–4; and Fig. 6C 1, 2 and 3), whereas the two SOD isoenzymes, isolated from strain 4C07 comprised a Cu-Zn/SOD (I) and a Fe/SOD (II) (Fig. 6B, SOD I and II, lanes 5–8; and Fig. 6C 4, 5 and 6).

The activity of SOD I in strain CC07, was reduced following incubation with ametryn and the two combined herbicides, whilst SOD II was unaffected (Fig. 6A, lanes 2 and 4, respectively). A different SOD isoenzyme pattern was detected following native PAGE of extracts of 4C07, in which SOD II activity was increased following treatment with clomazone and the two combined herbicides (Fig. 6B, lanes 7 and 8, respectively), whereas the activity of SOD I was also stimulated in the presence of clomazone, but drastically inhibited following the combined herbicide treatment (Fig. 6B, lanes 7 and 8, respectively). Ametryn did not produce any major change in SOD I and II activity when compared to the control (Fig. 6B, lanes 5 and 6).

**Figure 7:**
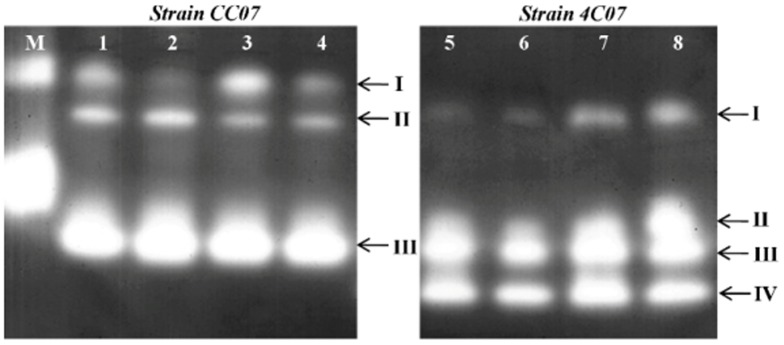
Activity staining for CAT following non-denaturing PAGE of extracts from cultured bacterial cells. Lane M is a bovine CAT standard, lanes 1, 2, 3 and 4, strain CC07 grown in the presence of 0 mM (control), 25 mM ametryn, 9 mM clomazone and 20 mM of each herbicide, respectively. Lanes 5, 6, 7 and 8, strain 4C07 grown in the presence of 0 mM (control), 25 mM ametryn, 9 mM clomazone and 20 mM of each herbicide, respectively. Arrows indicate sequentially numbered CAT bands for strains CC07 (I–III) and 4C07 (I–IV).

CAT activity staining following non-denaturing PAGE revealed the presence of three isoenzymes for strain CC07 (CAT I, II and III; Fig. 7, lanes 1–4) and four isoenzymes for strain 4C07 (CAT I, II, III and IV; Fig. 7, lanes 5–8), with CAT isoenzymes II (strain CC07), I (strain 4C07) and III (both bacteria) with the same relative mobility, suggesting they may be the same isoenzymes in both bacteria. The activity of CAT isoenzyme II was higher in 4C07 and was further increased when the strain was subjected to the combined herbicide treatment (Fig. 7, lane 8).

Furthermore, the CAT isoenzyme activity profiles were in a way similar to the enzyme pattern also observed for SOD (Fig. 6A and B). For instance, in strain CC07, the activity of CAT I was reduced in the ametryn and combined herbicide treatments (Fig. 7, lanes 2 and 4) as observed for SOD I (Fig. 6, lanes 2 and 4). On the other hand, the activity of the CAT II isoenzyme was slightly reduced in the clomazone and combined herbicide treatments (Fig. 7, lanes 3 and 4), whereas the CAT III isoenzyme was unaltered in all treatments. For strain 4C07, apart from the unique CAT II isoenzyme (Fig. 7, lane 8), the activity of CAT isoenzyme I was clearly increased following treatment with clomazone and the combined herbicides (Fig. 7, lanes 7 and 8).

**Figure 8:**
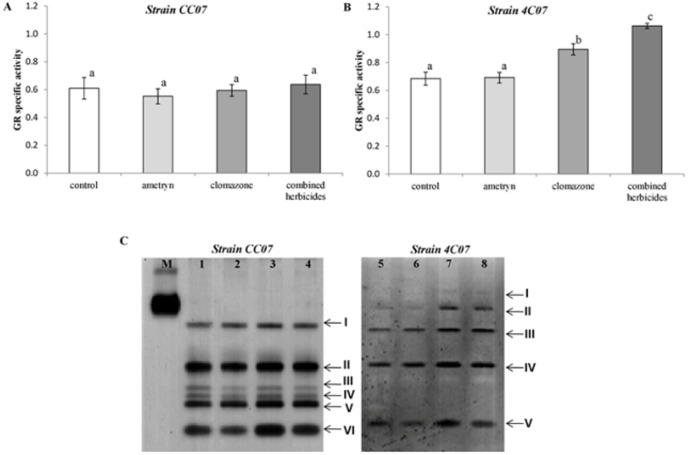
GR specific activity. (A) and (B) Specific activity of GR, expressed as µmol min^−1^ mg^−1^ protein. Values are the means of three replicates ±SEM. Means with different letters are significantly different (*P<*0.05) by one-way analysis of variance (ANOVA) and Tukey's test. (C) Activity staining for GR following non-denaturing PAGE of extracts of cultured bacterial cells. Lane M is a bovine GR standard, lanes 1, 2, 3 and 4, strain CC07 grown in the presence of 0 mM (control), 25 mM ametryn, 9 mM clomazone and 20 mM of each herbicide, respectively. Lanes 5, 6, 7 and 8, strain 4C07 grown in the presence of 0 mM (control), 25 mM ametryn, 9 mM clomazone and 20 mM of each herbicide, respectively. Arrows indicate sequentially numbered GR bands (I–VI) that are independent of the bacterial strain.

GR activity was determined as total specific activity (Fig. 8A and B) and by non-denaturing PAGE for isoenzyme identification (Fig. 8C). Total GR activity was not altered in strain CC07 regardless of the treatment (Fig. 8A), but was increased in strain 4C07 when exposed to clomazone (30.8% increase) and the combined herbicides (55.3% increase) (Fig. 8B). GR activity staining revealed the existence of 6 GR isoenzymes for strain CC07 (Fig. 8C, lanes 1–4), all present in all treatments tested, and up to 5 GR isoenzymes in the strain 4C07 depending on the treatment tested (Fig. 8C, lanes 5–8). In general, the GR isoenzyme activities were in accordance with the results obtained for total GR activity (Fig. 8A and B). Slight variations in band intensity were observed among the treatments, but all GR isoenzymes were present in the strain CC07 (Fig. 8C, lanes 1–4). On the other hand, there was increased total GR activity in strain 4C07, when subjected to clomazone and combined herbicide treatments, which is most likely due to the increased activity of GR isoenzyme II (Fig. 8C, lanes 7 and 8).

**Figure 9:**
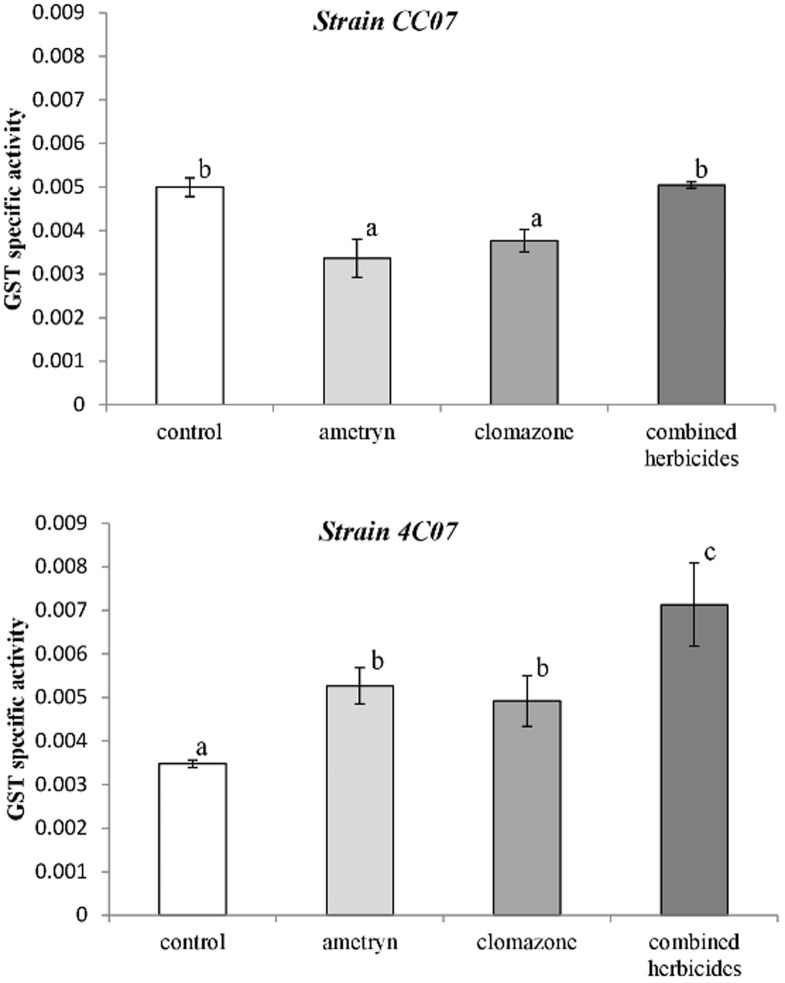
GST specific activity, expressed as units mg^−1^ protein. Values represent the means from three replicates ±SEM. Means with different letters are significantly different (*P<*0.05) by one-way analysis of variance (ANOVA) and Tukey's test.

Total specific GST activity was also determined and the results revealed a distinct response for each bacterium when exposed to the herbicides (Fig. 9). Total GST activity increased by 51.5%, 41.5% and 105% when strain 4C07 was grown in the presence of ametryn, clomazone or the combination of herbicides, respectively (Fig. 9). On the other hand, there was a reduction in GST activity when strain CC07 was exposed to the herbicides ametryn or clomazone, but the activity was similar to the control when CC07 was grown in the presence of the combined herbicides (Fig. 9).

## Discussion

Microorganisms present in soils must quickly adapt to environmental changes. This biochemical and physiological adaptation process play an important role in microbial survival, especially under stressful conditions [51]. Strains CC07 and 4C07 were selected for this study due to their ability to tolerate the high concentrations of herbicides tested and to the fact that bacteria of the *Pseudomonas* genus play a major role in degradation of xenobiotic compounds [52]–[55].

Generally, herbicides can alter the growth of degrading or tolerant bacteria [56]. Growth of the strains CC07 and 4C07 exhibited distinct responses when exposed to the herbicides ametryn and clomazone, separately or in combination. The number of bacterial cells for all treatments was lower when compared to the control, indicating that the ROS generated by the herbicides and its metabolites can damage bacteria cells and decrease bacterial growth. However, clomazone was the treatment that markedly affected growth of both strains. These results may be associated with the higher toxicity of the clomazone molecule, due the presence of chloride (Table 1). García-Cruz et al. [57] demonstrated the toxicity of the chlorine atoms present in the herbicide 2,4-dichlorophenoxyacetic acid (2,4-D) and possible intermediates in bacterial biofilms. Another possible cause of the aggressiveness of clomazone towards bacteria cells may be due to its mechanism of action, since it is an inhibitor of the deoxy-D-xylulose 5-phosphate (DXP) synthase enzyme [14], [58], which plays a key role in isoprenoid biosynthesis and is also required for thiamine and pyridoxal phosphate production in prokaryotes [59].

Previous studies have shown that when bacteria are exposed to herbicides or their metabolites, a significant increase in ROS generation may occur and, consequently, induce an oxidative stress condition [22], [23]. Lipid peroxidation is one of the best predictors of ROS level inducted under stress conditions [60], and can be used as a marker of oxidative stress [61]. An increased content of MDA was detected when strain CC07 was exposed to clomazone alone or in mixture with ametryn (Fig. 3), which suggests that the high concentration used in the combined treatment may have increased the amount of lipid peroxidation. Despite this, the CC07 strain was able to respond and tolerate the peroxidation stress, since following all the herbicide treatments, the growth observed after 14 h was very similar to that of the control (Fig. 2A). High concentrations of MDA have been found in strains of *Enterobacter asburiae* and *E. amnigenus* in the presence of the herbicides metolachlor and acetochlor, respectively [23], which correlated the oxidative stress with the mode of action of the herbicides tested, which involved the inhibition of the elongation of C18 and C16-fatty acids. On the other hand, changes in MDA content were not observed for strain 4C07 in the presence of the herbicides, suggesting that strain 4C07 could possess an effective antioxidant system to avoid the damage caused by ROS. Although no changes in MDA content were observed, the 4C07 strain exhibited limited growth, thus indicating sensitivity to the herbicides dose (s) in the medium.

To maintain ROS under the baseline levels, bacteria utilize a complex antioxidant defense system [20]. SOD is able to detoxify O_2_^−•^, one of the two substrates of the Haber-Weiss reaction, which generates OH^•^ radicals, and is therefore plays a key role in the central defense mechanism of living organisms [62]. Following analysis of SOD activity by non-denaturing PAGE, two isoenzymes were identified in each strain, which were classified as Mn-SOD (SODs I and II) for strain CC07, and as Cu/Zn-SOD (SOD I) and Fe-SOD (SOD II) for strain 4C07. Fe-SOD and Mn-SOD are present in the bacterial cytoplasm [63], whilst Cu/Zn-SOD isoenzymes are present in the periplasm and are more sensitive to endogenous oxidative stress. However, Cu/Zn-SODs are also responsible for protection against oxidative damage to DNA. Studies with mutants of *Escherichia coli* revealed that Cu/Zn-SOD activity increased bacterial resistance to the herbicide paraquat [64]. Thus, the presence of Cu/Zn-SOD in strain 4C07, in comparison to CC07, may have favored its antioxidant response to the O_2_^−•^ increase in the presence of the herbicides. In contrast, following exposure to the herbicide quinclorac, there was an increase in both Fe-SOD and Mn-SOD activity in the bacterium *B. cepacia* WZ [17], which appeared to protect *B. cepacia* from the redox action of quinclorac [17].

Catalases are widely distributed and are considered important components of detoxification routes that prevent the formation of hydroxyl radicals [65]. Three active isoenzymes (CAT I, III and IV) were detected in extracts of the untreated strain 4C07, however in the presence of both herbicides a new isoenzyme (CAT II) was induced. This may have occurred due to bacterial sensitivity to both herbicides, thereby, the expression of a new isoform would contribute to maintain the CAT activity at a standard level.

By comparing the CAT and SOD results for strain CC07, it can be seen that the herbicide ametryn inhibited the activity of both enzymes, (isoenzymes SOD I and CAT I in particular), whilst the herbicide clomazone had a greater effect on the enzymes of strain 4C07. There is no consensus on the effect of herbicides on CAT and SOD; both enzymes are essential in *Bacillus subtilis* B19, *B. megaterium* and *E. coli* K12 for survival in the presence of the herbicide bensulfuron-methyl [19], and both enzymes also played an important role in copper resistance in *Amycolatopsis* spp species [66]. In contrast, Martins et al. [23] reported that SOD activity was not significantly changed when strains of *E. asburiae* and *E. amnigenus* were exposed to the herbicides metolachlor and acetochlor, respectively.

Reduced glutathione is the most abundant thiol in cells and its main function is to maintain the cytoplasm redox state in equilibrium [29]. There was a significant increase in the GSH content of strain CC07 in the presence of ametryn, possibly minimizing the occurrence of lipid peroxidation. In the presence of clomazone alone or in mixture with ametryn, however the GSH content was reduced. Hultberg [67] observed decreases in the concentration of GSH when the bacterium *P. fluorescens* was exposed to cadmium and suggested that GSH may be used as a marker for the intensity of environmental stress. On the other hand, the GSH concentrations in strain 4C07 increased significantly in the presence of the herbicides. This result suggests a direct participation of GSH in ROS detoxification, particularly of the OH^•^ radical, consequently preventing lipid peroxidation and thus, contributing to the protection against oxidative stress. Generally in gram-negative bacteria, including *Pseudomonas*, the concentration of GSH is high [68] and it can react directly with a free radical resulting in a thiol radical, GS^•^, which reacts with another GS^•^ radical producing GSSG [69].

Under many intracellular stressful conditions the GSSG concentration increases due to GSH oxidation, however, a high GSH/GSSG rate is necessary to maintain the role of GSH as antioxidant and reducing agent [70], therefore, GR acts as a fundamental link between the two redox metabolites within a cell [29]. In strain CC07, total GR activity remained unchanged in the presence of the herbicides, which was reconfirmed with non-denaturing PAGE analysis. Veremeenko and Maksimova [68] showed that in *P. aurantiaca*, GSH and GR activity increased significantly in the presence of antibiotics. In this study, the increase in total GR activity in strain 4C07 in the presence of clomazone alone or in mixture with ametryn is associated with the increase in isoenzymes II and to a lesser extent I and V. These data suggest that the different isoenzymes may play a specific role in the antioxidant response of strain 4C07 to oxidative stress induced by the herbicides. Martins et al. [23] reported that when *E. asburiae* was exposed to the herbicide s-metolachlor (34 mM), two new GR isoforms were induced in response to the oxidative stress condition, suggesting a role for GR in herbicide tolerance. Similar responses were also found in plant species. For instance, in coffee cell suspension cultures subjected to the oxidative stress induced by heavy metals (cadmium, nickel and selenium) a new GR isoenzyme was induced during the first hours of stress. The authors also suggested that GR activity could be used as an early stress marker for this plant species [49], [71], [72].

GST is another enzyme with activity closely related to GSH. Its function is to conjugate GSH to xenobiotics, and therefore has a fundamental central role in detoxification [31]. GSTs are capable of detoxifying numerous classes of pesticides in bacterial cells including s-triazines and are involved in the first stage of the biodegradation of the herbicide atrazine, when the removal of the chlorine atom from the atrazine-GSH conjugate occurs [73]. The increase in GST activity in strain 4C07 may also be associated with the increase in GSH concentration, and could indicate the involvement of GST in the detoxification of the two herbicides.

**Figure 10:**
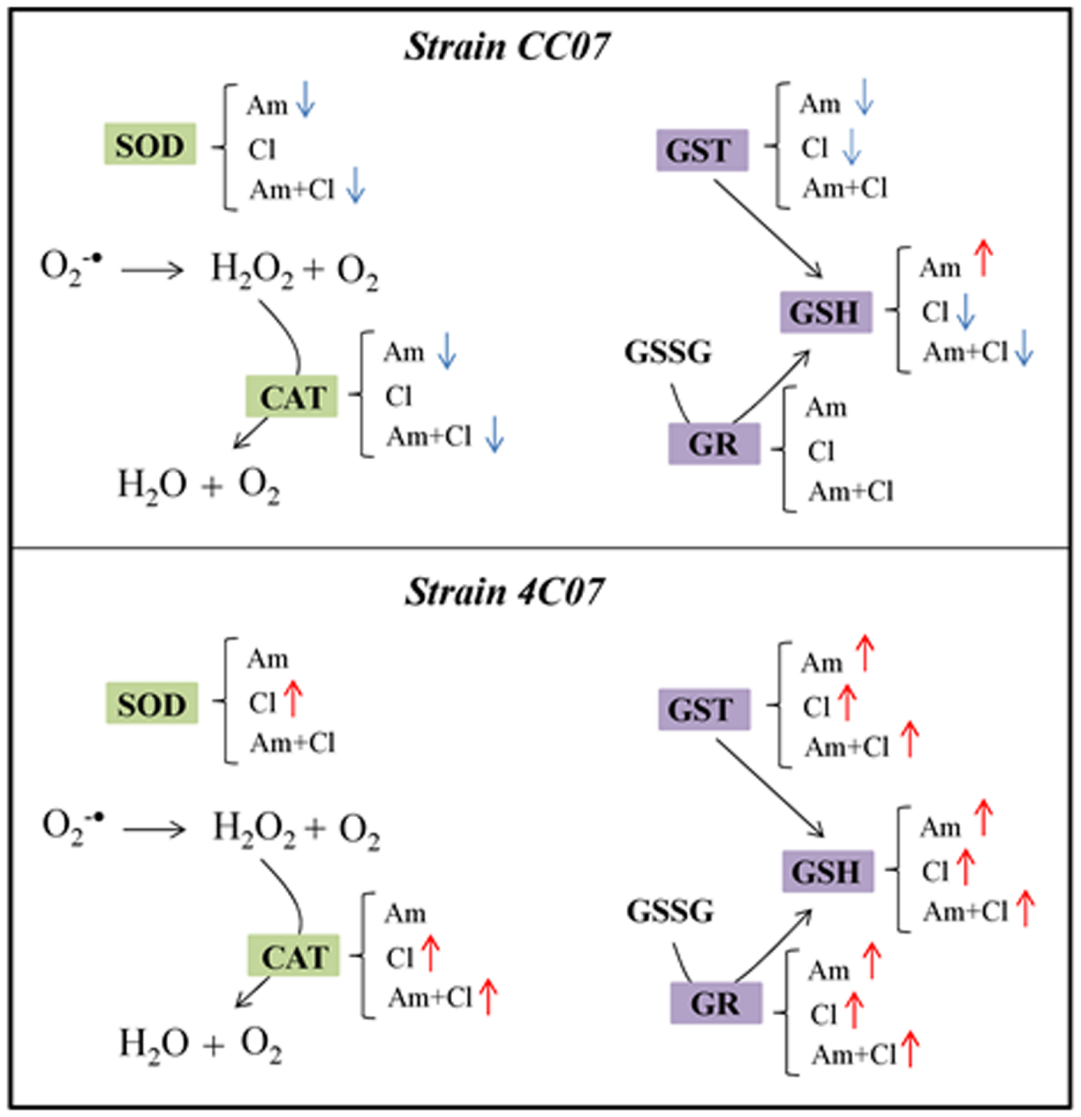
General view of the antioxidant system involving enzymatic and non-enzymatic components for the strains CC07 and 4C07 based on the data obtained in the present work. SOD (superoxide dismutase); CAT (catalase); GST (glutathione S-transferase); GR (glutathione reductase); GSSG (oxidized glutathione); GSH (reduced glutathione); Am (ametryn); Cl (clomazone); Am+Cl (combined herbicides). Blue arrows indicate decreases in enzymatic activity and glutathione content; red arrows indicate increases in enzymatic activity and glutathione content; the absence of symbols indicates no alterations. All changes are relative to the untreated control.

Our results indicate that both strains are tolerant to the herbicides ametryn and clomazone or to their mixture. However, the herbicides caused an imbalance in the redox potential and metabolism of the bacterial cells. A summary of the key alterations observed are presented in Fig 10. The enzymes GR and GST, together with the antioxidant compound GSH, may play a major role in the tolerance of strain 4C07 to the herbicides ametryn and clomazone supplied individually or combined. In contrast, there was a decrease in the activity of enzymes SOD, CAT, GST and the GSH content in CC07, whilst GR activity remained unchanged. Compensatory mechanisms for the reduction of these enzymes activities and the depletion of GSH content may occur and account for the induction of another defense mechanism, since the strain CC07 managed to adjust its metabolism in response to the stress. These results indicate a different antioxidant response of the two bacterial strains to the herbicides; however, additional studies are required in order to understand the tolerance mechanisms. Nevertheless, strain CC07 grew at a higher rate, indicating that this bacterium was able to adapt better to the stressful environment, which could be useful for bioremediation strategies of environments contaminated with herbicides.
